# Noninvasive Monitoring of Glycemia Level in Diabetic Patients by Wearable Advanced Biosensors

**DOI:** 10.3390/bios14100486

**Published:** 2024-10-08

**Authors:** Elena V. Daboss, Maria A. Komkova, Vita N. Nikitina, Egor A. Andreev, Darya V. Vokhmyanina, Arkady A. Karyakin

**Affiliations:** Chemistry Faculty, M.V. Lomonosov Moscow State University, 119991 Moscow, Russia; dabossev@my.msu.ru (E.V.D.); komkovama@my.msu.ru (M.A.K.); niki-vita@yandex.ru (V.N.N.); andreev@analyt.chem.msu.ru (E.A.A.); vokhmyaninadv@my.msu.ru (D.V.V.)

**Keywords:** diabetes, noninvasive, sweat, glucose, biosensor, Prussian Blue

## Abstract

We report on the possibility of noninvasive diabetes monitoring through continuous analysis of sweat. The prediction of the blood glucose level in diabetic patients is possible on the basis of their sweat glucose content due to the positive correlation discovered. The ratio between the blood glucose and sweat glucose concentrations for a certain diabetic subject is stable within weeks, excluding requirements for frequent blood probing. The glucose variations in sweat display allometric (non-linear) dependence on those in blood, allowing more precise blood glucose estimation. Selective (avoiding false-positive responses) and sensitive (sweat glucose is on average 30–50 times lower) detection is possible with biosensors based on the glucose oxidase enzyme coupled with a Prussian Blue transducer. Reliable glucose detection in just secreted sweat would allow noninvasive monitoring of the glycemia level in diabetic patients.

## 1. Introduction

The World Health Organization (WHO) reports on about 422 million people worldwide having diabetes. Diabetes is a chronic metabolic disease characterized by elevated levels of blood glucose, which over time leads to serious damage to the heart, blood vessels, eyes, kidneys, and nerves. The complications of diabetes (cardiovascular diseases, blindness, risk of amputation, kidney failure, etc.) can be postponed by maintaining the glucose concentration in the blood at an appropriate level.

Blood glucose is a rather flexible parameter; diabetics have to control it up to several times per day. This results in the annual market for personal glucose tests being > USD 15,000,000,000 [1]. To decrease the invasiveness of blood glucose analysis, implantable sensors stuck into fat tissues at about a 5 mm depth have been commercialized (see [2,3,4]). Despite these sensors being declared to analyze subcutaneous fluid, the detectable glucose levels are equal to them in blood [5,6].

Noninvasive methods exclude not only injury to blood vessels but also damage to the skin surface. They are painless and avoid potential infection and trauma to patients. However, physical methods were not successful; particularly, near-IR spectroscopy could not provide the required sensitivity and selectivity [7,8,9,10]. The attempts to deliver interstitial fluid to the skin surface by reverse iontophoresis for noninvasive glucose monitoring [11,12] were also unsuccessful in commercialized devices.

As a potential excreted liquid, we consider sweat [13]. It can be collected noninvasively after activation of the sweat glands by heating [14] or electrophoresis of pilocarpine [15,16]. For continuous monitoring of the sweat content, we have pioneered the flow-through system based on a biosensor integrated with a Macroduct-type sweat collector [13].

Obviously, not any excreted liquid can replicate blood in terms of the metabolite content. Indeed, there is no direct correlation between sweat and the blood glucose contents, either in healthy subjects [17,18] or in diabetics [18]. We, however, note that even in commercial implantable devices, the obtained values have to be corrected by specific algorithms [19]. We already claimed that the correlation in variation rates (ratios of glucose concentrations) is sufficient for diagnostics [17,20]. For healthy human subjects, we have shown positive correlation in the variation rates between the glucose concentrations in sweat and the corresponding values in blood [17]. The possibility of predicting blood glucose on the basis of its content in sweat has been confirmed using the touch-based fingertip self-testing protocol [21].

To validate the noninvasive monitoring of diabetes, it is necessary to discover such correlation for diabetics. An attempt has been made in [22]; however, the sweat samples were harvested by perfusion, obviously making the metabolite content dependent on the sweating rate, whereas the content of spontaneously secreted sweat is reported to be independent of it [23,24].

There is, however, a problem with the sensing part of a wearable device operated through sweat analysis. Since the glucose content in sweat as compared to blood is on average 30–50 times lower, with a minimum value of 5.6 µM [25], the corresponding biosensor sensitivity has to be two orders of magnitude higher than that of the whole blood or “subcutaneous fluid” analyzers.

The two monitors commercialized by Abbott use osmium complexes for low-potential glucose oxidation; however, they are based on glucose dehydrogenase [2], which has broader selectivity than glucose oxidase [26,27], thus providing a potential for false-positive readings, especially in sweat.

According to the patents assigned to Dexcom [28] and Medtronic [29], the biosensors in their implantable devices are based on platinum working electrodes. The proposed by G. Guilbault oxidation of hydrogen peroxide (H_2_O_2_), the side product of the glucose oxidase-catalyzed reaction, on platinum electrodes [30] is still operated in electrochemical clinical analyzers. However, for noninvasive monitoring, use of platinum as a transducer has the following problems. First, it is selective toward oxygen reduction rather than H_2_O_2_; hence, the latter can be detected exclusively by oxidation [31]. In the course of real sample analysis, this causes the appearance of false-positive signals due to the oxidation of reductants [32]. Second, in neutral physiological solutions, platinum becomes a poor electrocatalyst; the electrochemical constant below 7.5 × 10^−6^ cm s^−1^ [33] limits the H_2_O_2_ sensor’s sensitivity by the value of 7.5 × 10^−4^ A M^−1^ cm^−2^, which does not seem to be enough for sweat analysis.

Among the reported biosensors, the ones based on the most selective enzyme (glucose oxidase) and the sensitive low-potential H_2_O_2_ transducer are the most suitable for continuous analysis of excreted liquids (sweat). The ability to apply the corresponding advanced biosensors for noninvasive diabetes monitoring is provided by the discovered positive correlation between the glucose concentrations in sweat and the corresponding values in the blood of diabetic patients.

## 2. Materials and Methods

The experiments were carried out in distilled water. The inorganic salts, H_2_O_2_ (30 vol.%), D-glucose, and glucose oxidase (*Aspergillus niger*, 270 U·mg^−1^) were obtained from Sigma-Aldrich (Burlington, MA, USA). Moreover, the 10% perfluorosulfonated ionomer in isopropanol was obtained from Plastpolymer, and the pilocarpine (1%) from Diapharm (Saint Petersburg, Russia).

Electrophoresis was performed by Potok-1 (JSC “EMA PLANT”, Ekaterinburg, Russia). Sweat samples were collected with Macroduct (ELITech, Logan, UT, USA). The commercial glucometers were the FreeStyle Optium (Abbott, Green Oaks, IL, USA) and Contour TS/Contour Plus (Ascensia Diabetes Care, Leverkusen, Germany). The electrochemistry was investigated using the PalmSens4 potentiostat (PalmSens BV, Houten, The Netherlands). The screen-printed structures (Rusens, Moscow, Russia) had a carbon working electrode Ø = 1.8 mm. The platinum disk electrodes were based on Pt wire (Ø = 0.5 mm) pressed in Teflon. The flow-injection setup contained a wall-jet cell (nozzle Ø = 0.5 mm), which was connected to the injector (IDEX, Northbrook, IL, USA) and Perfusor Compact S pump (B. Braun, Melsungen, Germany).

Informed consent was obtained from all 22 diabetic subjects (12 females and 10 males, 18–69 years old). The experiments were carried out in accordance with GCP regulations. The experimental protocols were approved by the Ethical Committee of Pulmonology Research Institute (Moscow, Russia). Prior to the sample collection, the subjects were wiped and cleaned with an alcohol swab and deionized water on the skin area of interest. The sweat sample collection was carried out according to a clinically relevant procedure based on the stimulation of the sweat glands through electrophoresis of 1% pilocarpine for 15 min. The capillary blood glucose was measured with at least 3 glucose test strips 15 min after the start of sweating.

The glucose in the sweat samples, diluted 10–15 times with 150 mM phosphate buffer (pH 6.0) containing 500 mM NaCl, was detected using a flow-injection analyzer equipped with a Prussian Blue-based glucose biosensor; the protocol had been validated with EcoBasic (Care Diagnostica, Voerde, Germany) [17].

The glucose biosensors were produced by drop-casting 2 μL of mixture (1 mg·mL^−1^ of glucose oxidase in perfluorosulfonated ionomer: 0.3%, pH 5.5) onto the Prussian Blue-modified or Pt electrode. The biosensors were operated at E_DC_ = 0.0 V for Prussian Blue and E_DC_ = 0.6 V for the Pt-based transducers.

## 3. Results and Discussion

### 3.1. Sweating by Diabetics

There is an accepted view that the skin surface of diabetics is dryer as compared to healthy subjects. Hence, sweat sampling from diabetic patients has to be addressed first. The most widely used clinically relevant procedure for noninvasive sweat collection (electrophoresis of pilocarpine) has been chosen.

**Figure 1:**
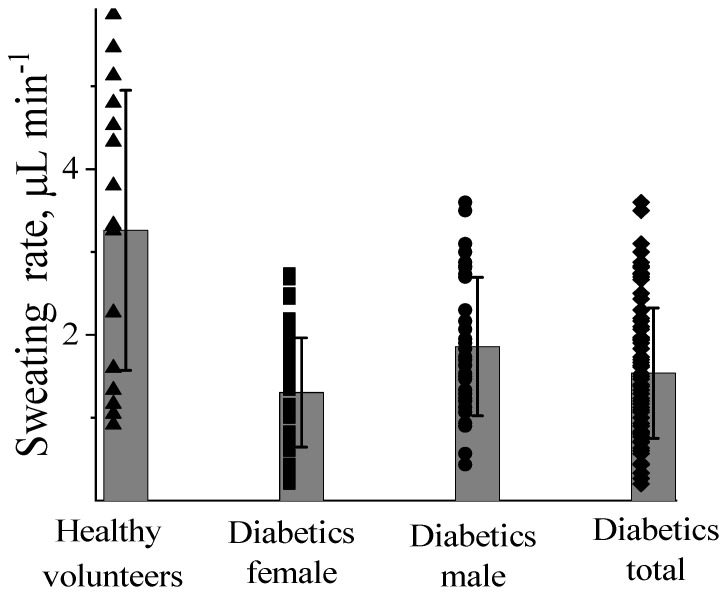
Sweating rates (filled marks) of healthy subjects (16) and female (48) and male (35) diabetics; columns with error bars represent the mean values for each set of data.

The sweating rate has been determined by dividing the collected sweat volume by the sweating time. Figure 1 displays the sweating rates measured several times for 22 diabetics, among them 10 men and 12 women, as well as for healthy subjects. As can be seen, the sweating rate of the diabetics is generally lower as compared to the healthy subjects. Similarly to the healthy subjects, the male diabetics are sweating more intensively than the female diabetics. However, even the lowest rate detected for a female diabetic subject (0.2 µL min^−1^) is, for example, at the upper range of the insulin pump [34]. Moreover, the range of diabetics’ sweating rates overlaps that of healthy subjects (Figure 1). We are, thus, able to conclude that the sweating rates of diabetics are sufficient to ensure the operation of wearable devices.

### 3.2. Relation between Sweat and Blood Glucose Contents in Diabetics

Prior to analysis of the sweat samples, a flow-injection system equipped with a Prussian Blue-based glucose biosensor has been characterized. The calibration is linear, with sensitivity of 22 ± 2 mA·M^−1^·cm^−2^, which is typical for such systems, and the detection limit is 2.1 ± 0.3 μM. The response to diluted sweat samples always belonged to the linear calibration range of the system (Figure S1).

As mentioned, the key problem for diabetic patients is monitoring the glucose concentration in their blood, which requires frequent blood probing. Hence, noninvasive approaches should allow the estimation of the blood glucose concentration.

The first question is whether sweat’s metabolite content is somehow dependent on the sweating rate. The glucose concentration in the sweat samples collected from 22 diabetics varied from 25 µM to 440 µM (Figure S2). Plotting the sweat glucose against the reciprocal sweating rate (Figure S2), we have not found any correlation (Pearson coefficient r = 0.346). It is thus possible to conclude that the sweat glucose content in diabetic patients is independent of the sweating rate. Quite a similar conclusion concerning the lactate content in the sweat of healthy subjects can be found [23,24].

As mentioned, it is not feasible to expect a direct correlation between blood and sweat. Indeed, plotting the absolute values of the glucose content in these biological liquids against each other, we have not been able to discover any correlation (Figure S3): the Pearson coefficient found has even been below 0.1 (r = 0.096). A similarly unsatisfactory result has been reported in [18].

As we supposed earlier [20], if occasional blood probing for calibration does not appear to devalue a diagnostic tool referred to as “noninvasive”, a sufficient requirement would be a correlation in the variation rates between the metabolite concentrations in the excreted liquid and the corresponding values in the blood. We determine the variation rate as the ratio of the glucose concentration measured on different occasions.

**Figure 2:**
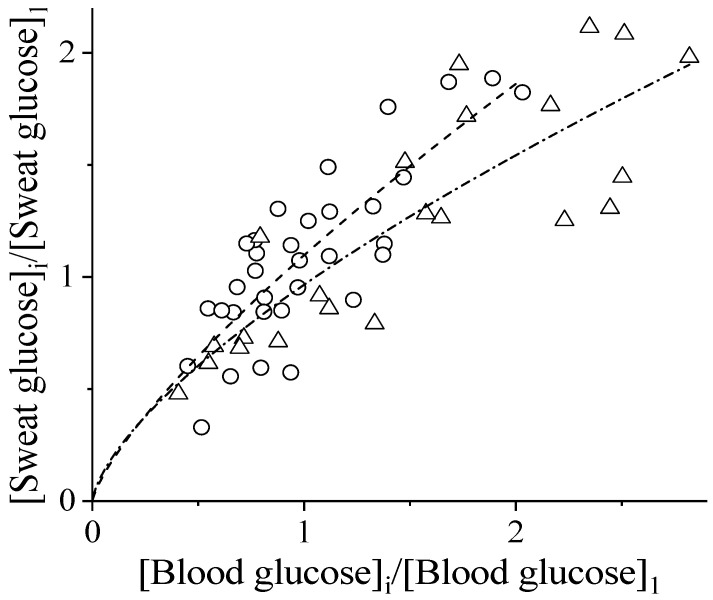
Ratios of the glucose concentrations in the sweat and blood of diabetic patients relative to the first measurement for the female (○) and male (∆) subjects; fitting to the allometric power function for the female (dash) and male (dash–dot) curves.

Figure 2 presents the variation rates for the sweat glucose vs. those for the blood glucose in 22 diabetics (12 female and 10 male subjects). The first measurement for each patient has been taken as the reference. As can be seen, the shape of the data cloud points to correlation. Indeed, the Pearson correlation coefficients for the male subjects reached the value of r = 0.85, and for the female diabetics, r = 0.82. The Pearson correlation coefficient for all the points is r = 0.81, pointing to slightly different functions for the male and female subjects. We note that the correlation coefficients achieved for the diabetic patients are even higher than those found by us for healthy subjects [17]. This confirms the possibility of monitoring diabetes through continuous analysis of sweat.

An important issue arises: how to recalculate the sweat glucose to blood glucose levels. The observed correlation (see Figure 2) points to the direct proportionality between the sweat and blood glucose concentrations with a zero free term. However, linear regression of the data in Figure 2 would return a non-zero free term.

If the sweat glucose is the linear function of the blood glucose with a non-zero free term, the differential variation rates should be studied. In other words, instead of the ratio fif1, as in Figure 2, the ratios of type (fi−f1)(fj−f1) should be used. The difference in function values ($f_{i}$) would obviously neglect the free term. For this aim, the ratios of the blood glucose and sweat glucose contents have been generated (Figure S4). However, despite the regression line seeming to cross the origin, such generation significantly decreases the Pearson correlation coefficient. Hence, the addition of a non-zero free term to the linear dependence between the variation rates of the sweat and blood glucose, on the contrary, deteriorates the regression.

Another possibility to improve the fitting of the dependencies in Figure 2 is through the non-linear dependence between sweat and blood glucose. Indeed, fitting to the allometric power functions ($y=a\cdot x^{b}$) improves the regression in terms of the decreased residual sum of squares. The exponent (b) for the female subjects has been found to be 0.76 (dashed curve); for male subjects, of 0.68 (dash–dot curve, Figure 2). Importantly, coefficient “a” for both the male and female subjects is almost equal to unity. No indication of a free term in the allometric function has been found.

Accordingly, evaluation of the blood glucose (*BG*) on the basis of the sweat glucose (*SG*) content is possible through Formula (1):

(1) $${BG}_{i}={BG}_{1}{\left(\frac{{SG}_{i}}{{SG}_{1}}\right)}^{z}$$

where the lower index indicates the first (1) and current (*i*) measurements, and *z* is the exponent, which can be taken as equal to unity or, more precisely, to 1.3 (1/0.76) for the female diabetic subjects and to 1.4 for the male ones. Obviously, for every subject, the exponent can be evaluated individually during the first days of monitoring. We note that except for the Abbott Libra, all the low-invasive implantable devices require frequent (daily) calibration vs. blood.

**Figure 3:**
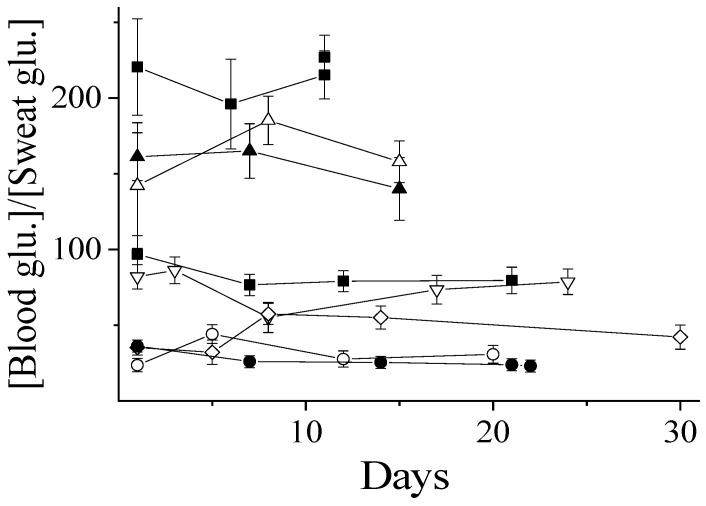
Time dependencies of the blood-to-sweat glucose ratios for several diabetic subjects (each symbol indicates a particular diabetic subject).

The frequency of the recalibration for a certain diabetic subject depends on how stable the ratio of blood-to-sweat glucose is. As can be seen in Figure 3, this ratio can be varied within an order of magnitude from 20 to 220. However, for a particular diabetic subject, the ratio of blood-to-sweat glucose has been found to be stable with the precision of measurements (Figure 3), despite the time frame of the investigations lasting up to 30 days in some cases. It is thus possible to conclude that the blood-to-sweat glucose ratio is stable up to a period of weeks, and it is not necessary to recalibrate the blood glucose content against the sweat levels upon operation of a single wearable device.

### 3.3. Glucose Biosensors for Continuous Sweat Analysis

Another problem to be addressed is the sensing principle. Since the sweat glucose content is much lower as compared to that of the blood (above), the most selective enzyme (glucose oxidase) should be used. Considering the above discussion, the most suitable biosensor for continuous sweat monitoring is the first generation one based on an H_2_O_2_ transducer.

We have carried out a direct comparison of glucose biosensors based on different transducers. As mentioned, four out of six implantable low-invasive electrochemical monitors [3], namely those produced by Medtronic and Dexcom, are based on platinum working electrodes. Low-potential H_2_O_2_ detection has been found to be impossible using Pt. The noticeable sensitivity of the corresponding glucose biosensor (12 mA·M^−1^·cm^−2^) is only achieved at a working electrode potential of +0.6 V vs. Ag|AgCl. At this potential, however, the sensitivities to ascorbate, uric acid and paracetamol are 10 times higher than to glucose (Figure S5). Since the median of the glucose content in sweat is only three times higher than that of uric acid [25], the false-positive response to the latter would exceed 300%. We note that an erroneous decrease of blood glucose is dangerous, because while 3.9 mM is normal (https://www.who.int/data/gho/indicator-metadata-registry/imr-details/2380), accessed on 1 September 2024 coma can occur from 2.7 mM [35].

**Figure 4:**
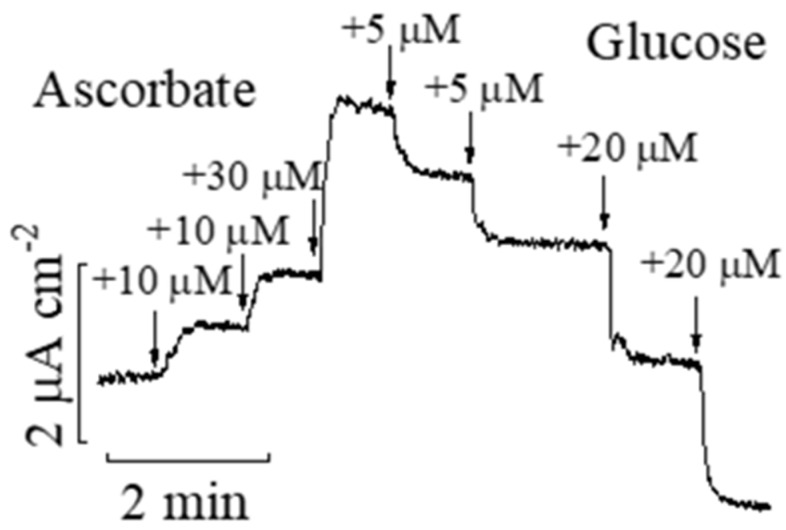
Responses of the Prussian Blue-based glucose biosensor to ascorbate and glucose.

The sensitivity of the Prussian Blue-based glucose biosensor is five times higher than that with a platinum transducer (Figure S6). Concerning its selectivity, first, the responses to reductants and to glucose are oppositely directed (Figure 4). Hence, the reductants cannot in principle generate a false-positive signal in Prussian Blue-based biosensors. Second, displaying negligible (two orders of magnitude lower) responses to uric acid and paracetamol, the biosensor demonstrates a twice lower response to ascorbate as compared to glucose (Figure 4). The median of the ascorbate concentration in sweat is 17 times lower than the glucose content [25]. Hence, the Prussian Blue-based transducer solves the problem of reductants in the course of glucose detection also in sweat, because even the most dangerous one may generate an error of less than 3%.

**Figure 5:**
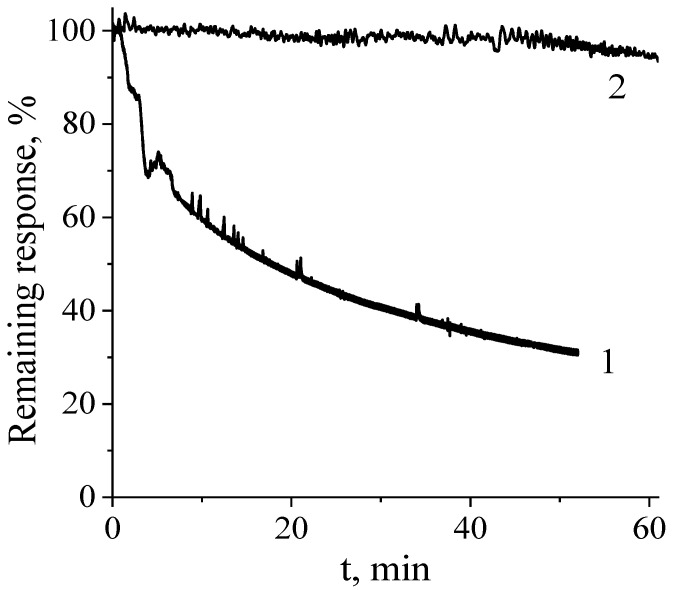
Remaining response of the Pt-based (1) and Prussian Blue-based (2) glucose biosensors toward 250 µM glucose upon monitoring of undiluted human sweat.

Another problem in terms of noninvasive diabetes monitoring through continuous sweat analysis is the transducer stability. Since sweat contains a variety of oligopeptides (including ones containing sulfide substituents), noble metal-based catalysts are expected to be poisoned in this biological liquid. Indeed, after 1.5 h of incubation of the platinum-based glucose biosensor in human sweat, its sensitivity decreases 10 times (Figure S7). On the contrary, the sensitivity of the Prussian Blue-based glucose electrode remains at the level of 80%, even after 2.5 h (Figure S7). We display the behavior of the corresponding glucose-sensitive electrodes upon monitoring 250 µM glucose in undiluted human sweat in Figure 5. As can be seen, after one hour, the response of the Pt-based biosensor is decreased down to 20%, whereas use of Prussian Blue as a transducer provides more than 90% of the response. Accordingly, the latter is advantageous over noble metal-based transducers providing monitoring of undiluted sweat with the corresponding biosensors.

We have already reported on continuous glucose monitoring in just secreted sweat [17]. This is possible with a flow-through biosensor [13] on the basis of a Macroduct-type sweat collector applied to the skin surface and thus can be referred to as a wearable device. Here, we are confirming this possibility with the use of Prussian Blue-based glucose biosensors. This is shown by comparing the readings of the flow-through biosensor directly applied to the skin surface with the results of the independent sweat analysis performed from the outlet of the monitor (Figure S8).

## 4. Conclusions

We report on noninvasive diabetes monitoring by evaluating the blood glucose concentration on the basis of the glucose content in the sweat of patients with diabetes mellitus. This is possible after calibration, i.e., estimation of the ratio between the blood glucose and sweat glucose contents. This ratio has been found to be stable within weeks, excluding the frequent blood probing required for the majority of low-invasive implantable devices.

Platinum-based biosensors are hardly applicable for continuous sweat analysis. First, their sensitivity in undiluted sweat is continuously decreased down to 10 times, becoming two orders of magnitude lower as compared to Prussian Blue-based ones, which is not enough for diabetes monitoring. Second, Pt-based glucose biosensors suffer from low selectivity relative to reductants: in sweat, the false-positive response to uric acid would achieve 300%. On the contrary, the selective and sensitive low-potential H_2_O_2_ transducer (Prussian Blue) is suitable for whole sweat analysis and allows successful operation of a noninvasive, wearable diabetes monitor.

## Data Availability

Data are provided within the article or Supplementary Materials.

## Supplementary Materials

The following supporting information can be downloaded at https://www.mdpi.com/article/10.3390/bios14100486/s1, Figure S1: Responses toward model glucose solutions; Figure S2: Glucose content in the sweat of diabetic patients as a function of the sweating rate; Figure S3: Relation between the sweat and blood glucose of diabetic patients; Figure S4: Generated ratios of the blood glucose and sweat glucose contents; Figure S5: Calibration graphs for the glucose biosensors based on Pt; Figure S6: Calibration graphs for the glucose biosensors based on Prussian Blue and on Pt; Figure S7: Retaining the sensitivity of biosensors based on Pt and Prussian Blue in human sweat. Figure S8: Readings of the noninvasive monitor vs. the glucose content in sweat.
