# Supplementary material for: Noninvasive Monitoring of Glycemia Level in Diabetic Patients by Wearable Advanced Biosensors
Source: Biosensors (Basel). 2024 Oct 8;14(10):486. doi: 10.3390/bios14100486 (PMC11505642; doi:10.3390/bios14100486)
Supplement: Supplementary file 1 [file biosensors-14-00486-s001.zip › biosensors-3215343-supplementary.pdf]

biosensors

Suplemmentary Materials

Noninvasive Monitoring of Glycemia Level in Diabetic Patients by Wearable Advanced Biosensors

Elena V. Daboss, Maria A. Komkova, Vita N. Nikitina, Egor A. Andreev, Darya V. Vokhmyanina and Arkady A. Karyakin *

Chemistry faculty of M.V. Lomonosov Moscow State University, 119991 Moscow, Russia; dabossev@my.msu.ru (E.V.D.); komkovama@my.msu.ru (M.A.K.); niki-vita@yandex.ru (V.N.N.); andreev@analyt.chem.msu.ru (E.A.A.); vokhmyaninadv@my.msu.ru (D.V.V.)

* Correspondence: aak@analyt.chem.msu.ru

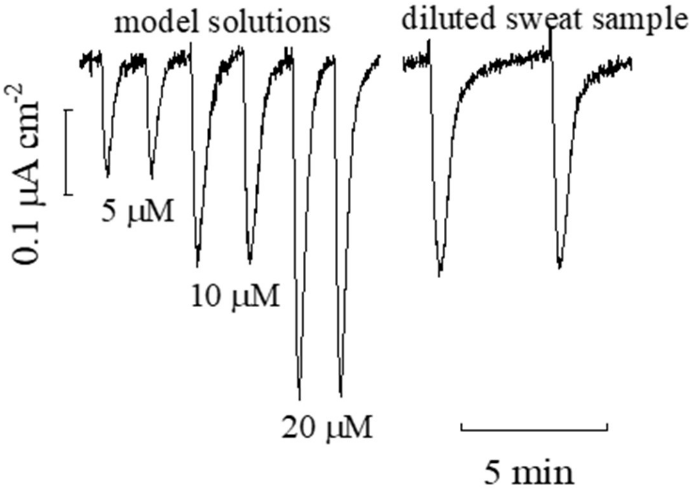

Figure S1. Responses towards model glucose solutions and diluted sweat sample registered in the flow-injection amperometry regime; 30 ml·h^{-1} flow rate, 150 mM phosphate buffer (pH 6.0) containing 500 mM NaCl.

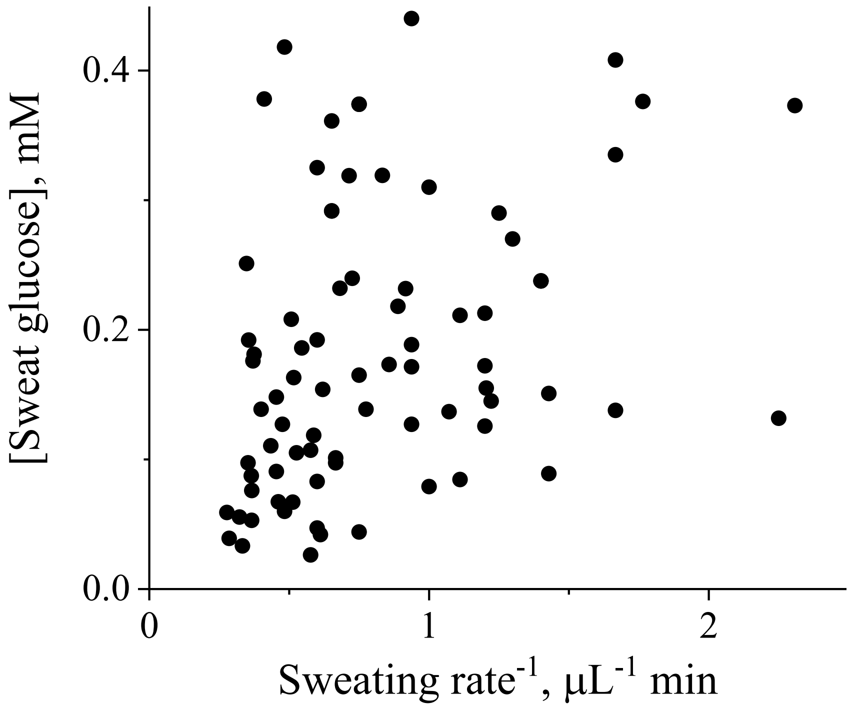

Figure S2. Glucose content in sweat of diabetic patients as a function of sweating rate.

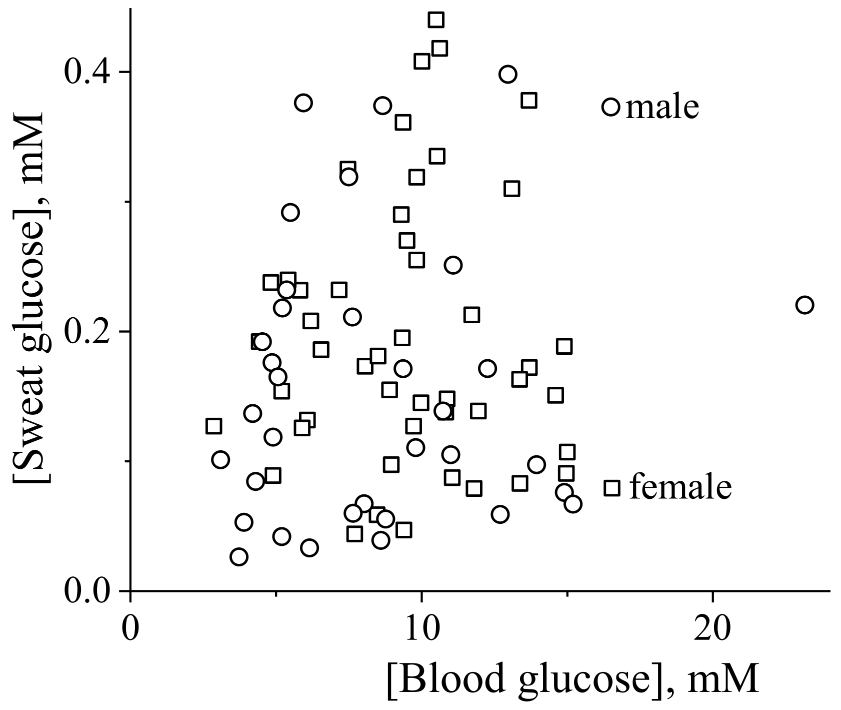

Figure S3. Relation between sweat and blood glucose of diabetic patients: (○) – male and (□) – female subjects.

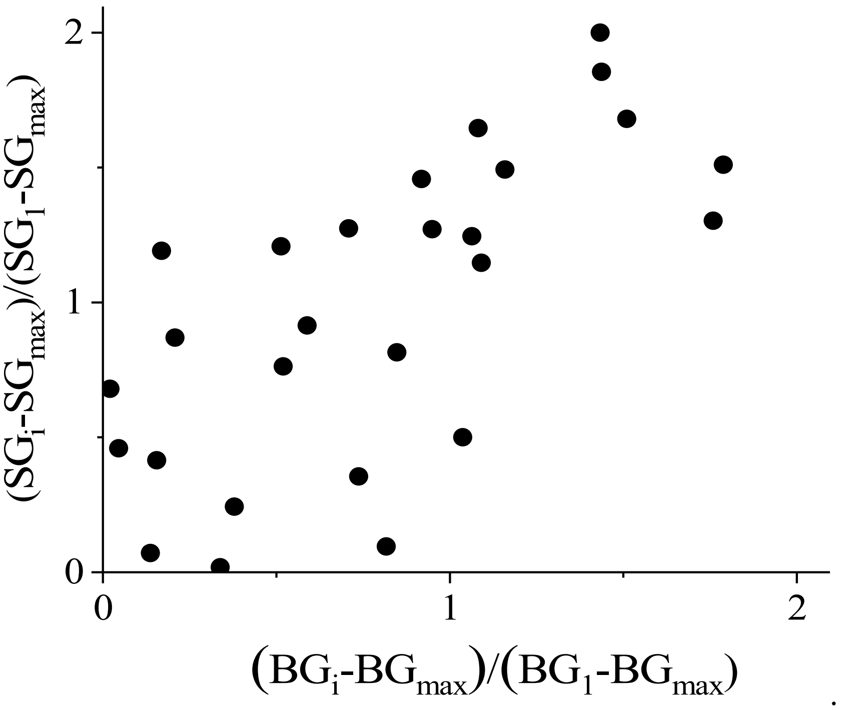

Figure S4. Generated ratios of blood glucose (BG) and sweat glucose (SG) contents with fixed first and maximum values.

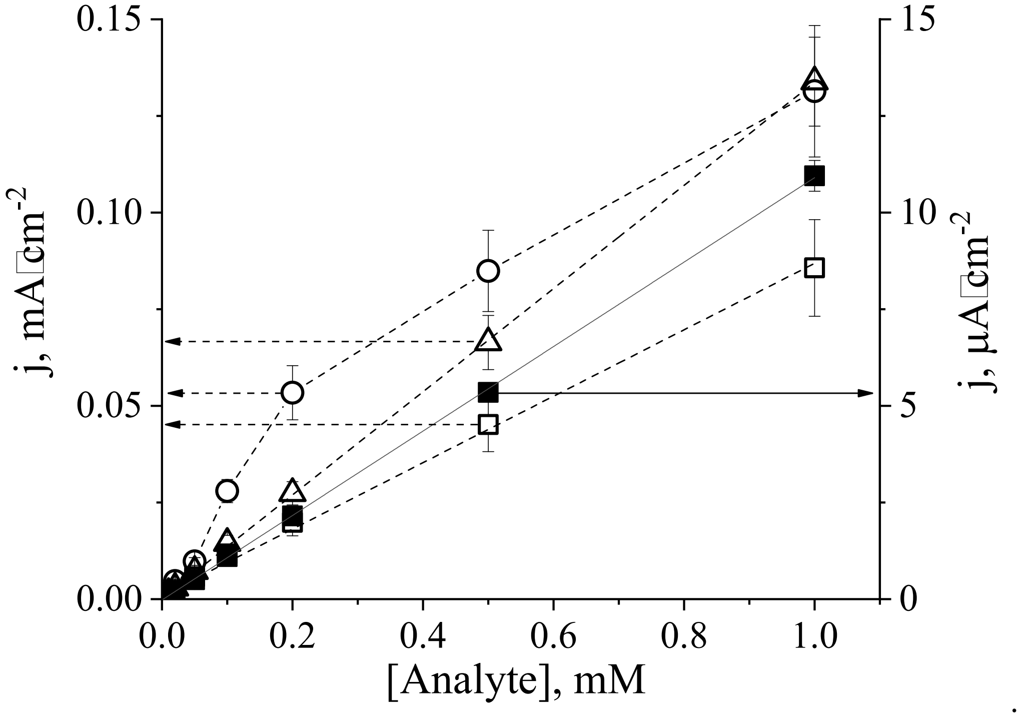

Figure S5. Calibration graphs for glucose biosensors based on glucose oxidase immobilized in Nafion on Pt disc electrode towards: ascorbate (Δ), paracetamol (□), uric acid (○), and glucose (■); E_{DC}=0.6 V.

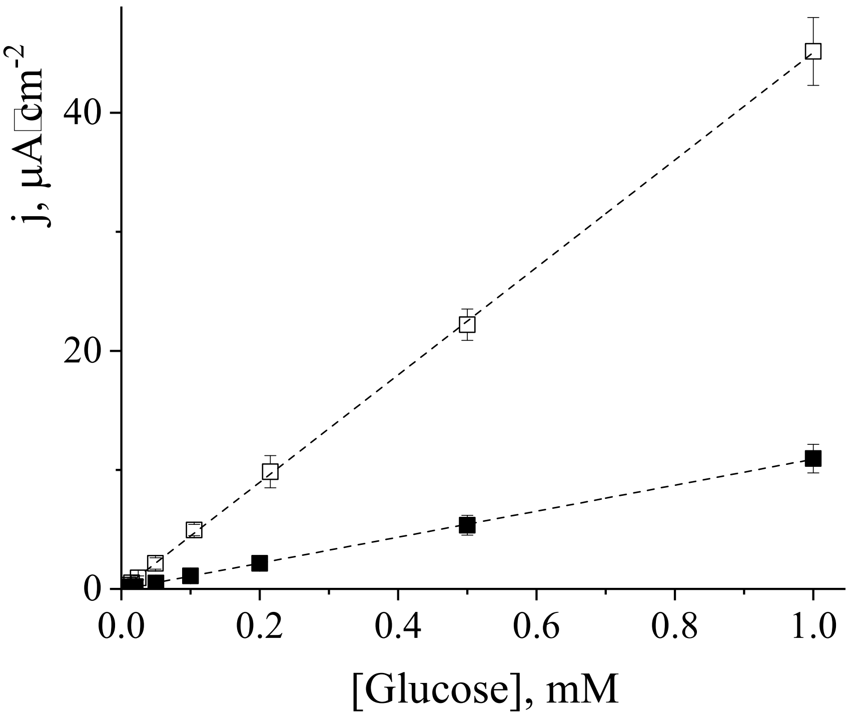

Figure S6. Calibration graphs for glucose biosensors based on glucose oxidase immobilized in Nafion over Prussian Blue (□, E_{DC}=0.0 V) and on Pt disc electrode (■, E_{DC}=0.6 V).

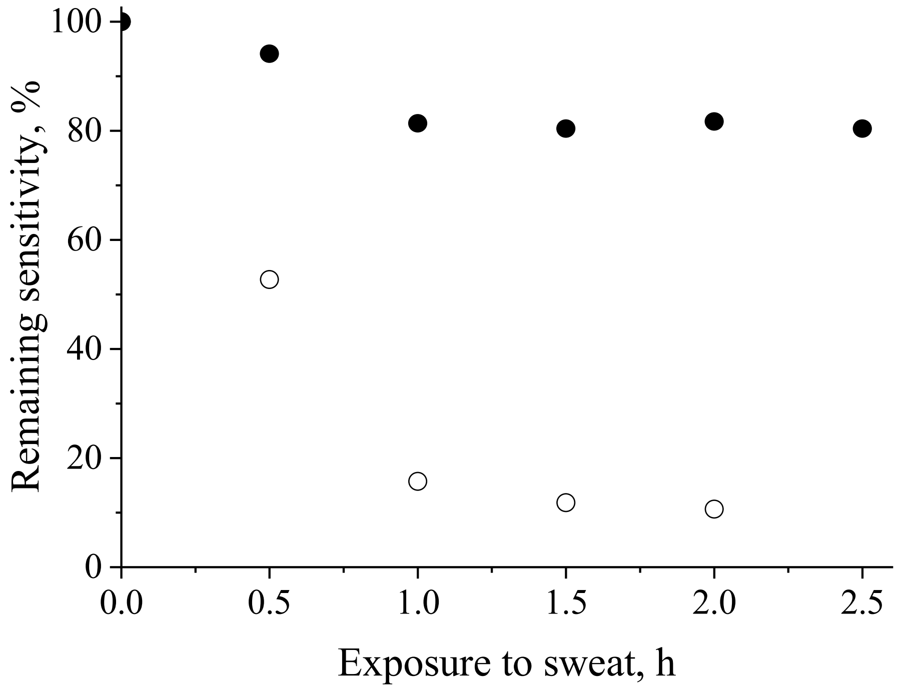

Figure S7. Remaining sensitivity of glucose biosensors based on platinum (○) and Prussian Blue (●) upon exposure to undiluted human sweat.

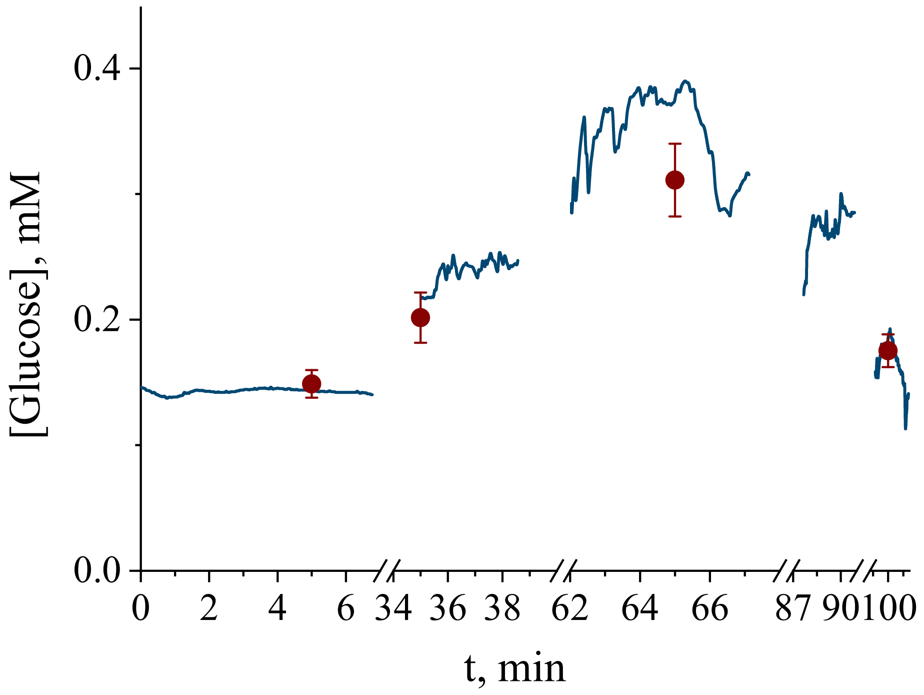

Figure S8. Readings of non-invasive flow-through monitor (line) and independently evaluated glucose content in sweat, collected from the outlet of the monitor (●).
